# Microstructural Evolution of AlCoCrFeNiSi High-Entropy Alloy Powder during Mechanical Alloying and Its Coating Performance

**DOI:** 10.3390/ma11020320

**Published:** 2018-02-23

**Authors:** Lihui Tian, Ming Fu, Wei Xiong

**Affiliations:** National Demonstration Center for Experimental Materials Science and Engineering Education (Jiangsu University of Science and Technology), Zhenjiang 212003, China; mingfu_2016@163.com (M.F.); xiongwei32410@163.com (W.X.)

**Keywords:** AlCoCrFeNiSi high-entropy alloy (HEA), coating, mechanical alloying (MA), atmospheric plasma spraying (APS), microstructural evolution, microhardness, wear behavior

## Abstract

High-entropy alloys (HEAs) are promising structural materials due to their excellent comprehensive performances. The use of mechanically alloyed powders to deposit HEA coatings through atmospheric plasma spraying (APS) is an effective approach that can broaden the application areas of the HEAs. In this paper, a ductility–brittleness AlCoCrFeNiSi system was chosen as an object of study, and the detailed evolution of the surface morphology, particle size distribution, and microstructure of the powder during mechanical alloying was investigated. An AlCoCrFeNiSi HEA coating was deposited using powder milled for 10 h, which can be used as an ideal feedstock for APS. The surface morphology, microstructure, microhardness, and wear behavior of the coating at room temperature were investigated. The results showed that as the milling time increased, the particle size first increased, and then decreased. At the milling time of 10 h, simple body-centered cubic (BCC) and face-centered cubic (FCC) solid solution phases were formed. After spraying, the lamellar structure inside a single particle disappeared. An ordered BCC phase was detected, and the diffraction peaks of the Si element also disappeared, which indicates that phase transformation occurred during plasma spraying. A transmission electron microscopy analysis showed that nanometer crystalline grains with a grain size of about 30 nm existed in the APS coating. For the coating, an average microhardness of 612 ± 41 HV was obtained. Adhesive wear, tribo-oxidation wear, and slight abrasion wear took place during the wear test. The coating showed good wear resistance, with a volume wear rate of 0.38 ± 0.08 × 10^−4^ mm^3^·N^−1^·m^−1^, which makes it a promising coating for use in abrasive environments.

## 1. Introduction

High-entropy alloys (HEAs), which are composed of five to 13 main elements, with each element’s content 5–35 at %, have been studied for more than 10 years, ever since they were first defined in 2004 [1]. Usually, HEAs are composed of either single or multiple body-centered cubic (BCC) or face-centered cubic (FCC) phases, and most of the investigations reported have focused on single phase alloys [2]. Recently, a dual phase AlCoCrFeNi HEA was prepared by arc melting and homogenization heat treatment [3]. Results showed that the precipitation of the FCC phase at the BCC grain boundaries reflected the effect of the FCC phase on crack deflection and branching during propagation under tensile loading. It was found that the presence of a ductile FCC high-entropy phase can impart good room temperature ductility to the brittle BCC phase. The excellent comprehensive performance, which includes its high strength, high hardness, good wear resistance, and good thermal stability, make HEAs promising industrial structural materials that can replace the traditional ones in the future [4,5,6].

Until now, many processes have been used to prepare bulk HEA materials [1,5,7,8]. Although bulk HEAs possess good properties, the high cost, which is caused by the use of a large number of high-purity elemental materials, limits their industrial applications. As is well known, coating deposition is an effective approach to broaden the application areas of high-cost materials due to its material saving [9]. So far, some technologies, such as cathodic arc vapor deposition (CAVD) [10], magnetron sputtering [11], and laser cladding [12,13] have been employed to deposit HEA coatings. However, some drawbacks restrict the application of these technologies, such as the high cost and low deposition efficiency for magnetron sputtering, and the high residual stress and high dilution for laser cladding [14,15]. Since it is different from the above technologies, atmospheric plasma spraying (APS) is an appropriate technology for depositing HEA coatings, as those drawbacks can be overcome. By now, some investigations on APS HEA coatings have been carried out [16,17,18], in which coatings with good properties were obtained. In addition, in the authors’ previous study [18], an AlCoCrFeNiTi HEA coating was deposited by APS using mechanically alloyed (MA) powder as a feedstock, and it exhibited outstanding properties, such as high bonding strength, high hardness, and excellent wear resistance.

Among the preparation methods of the feedstock powders for APS, mechanical alloying is a frequently used one, owing to its simple, continuous, and controllable process and low cost [19], and it is usually used to synthesize amorphous alloy, supersaturated solid solution, and intermetallic compounds [19]. So far, very few investigations have been reported on the preparation of HEA coatings by APS using mechanically alloyed powders. For example, nanostructured MnCoCrFeNi and AlCoCrFeNi high-entropy alloy coatings were deposited by APS with mechanically alloyed powders [16]. However, the MA process, in which the microstructure and properties of the powders can change significantly with the milling time, was not investigated in detail. Furthermore, lots of papers focused on the evolution of the particle size and the phase structure of HEA powders during mechanical alloying, but without a coating deposition [20,21,22,23]. In these papers, different alloy systems composed of common elements such as Al, Co, Cr, Cu, Fe, Mn, Ni, Ti, and Zn were chosen as the objects of study. The results showed that the chemical composition of the powders has a significant effect on their evolution processes. Still, some other important characteristics, such as the cross-sectional microstructure and the hardness of the powders, which can also change with the milling time, were not investigated. As a feedstock powder for APS, the mechanically alloyed powder should be with some appropriate properties such as shape, particle size distribution, flowability, cross-sectional microstructure, and phase structure, which are key influencing factors to the coating quality, and should be further investigated.

Usually, in an HEA system, composition of the elements can impact on the alloy’s behavior, phase assemblages, and properties [24]. In general, transition metal elements of the fourth period are used as the matrix elements, and some other alloying elements, especially with a larger atomic size difference, are added into the matrix in order to enhance the mechanical properties such as strength, hardness, and plasticity by changing the lattice distortion and the lattice types. For example, it was reported that the Al element, with its larger atomic radius, has an effect on the microstructure and properties of Al*_x_*CoCrFeNi by vacuum arc melting and casting methods [25], and with the increase of Al content, the formation of the body-centered cubic (BCC) phase was enhanced, and the hardness increased. Al_0.5_CoCrCuFeNiSi*_x_* alloys were prepared by arc melting, and results showed that with an increase in Si content, the microstructure of the alloys changed from face-centered cubic (FCC) to BCC, the compressive strength increased, and the ductility decreased [24]. It was also reported that for vacuum arc melted AlCoCrNiSi*_x_* alloys, with an increase in Si content, the phase structure was transformed from a single BCCl structure to a mixed BCCl + BCC2 structure, and the hardness increased [26].

Therefore, in this paper, an AlCoCrFeNiSi system was chosen as an object of study, in which Al and Si elements were added into the CoCrFeNi matrix in order to enhance its performance. In the AlCoCrFeNiSi system, elements such as Al, Co, Cr, Fe, and Ni are ones with good ductility, while Si is a brittle element. For such a ductility–brittleness system, the evolution of the characteristics of the powder during mechanical alloying must be quite complex and different from the ductility–ductility ones mentioned above [20,21,22,23]. The detailed evolution of the surface morphology, particle size distribution, cross-sectional microstructure, and phase structure of the powder during mechanical alloying was investigated. In addition, an AlCoCrFeNiSi HEA coating was deposited by APS using the mechanically alloyed powder. The variation of the cross-sectional microstructure and the phase structure before and after spraying was analyzed. The microhardness and the wear behavior of the APS coating at room temperature were also investigated.

## 2. Experimental

### 2.1. Preparation of the AlCoCrFeNiSi HEA Powder

**Figure 1:**
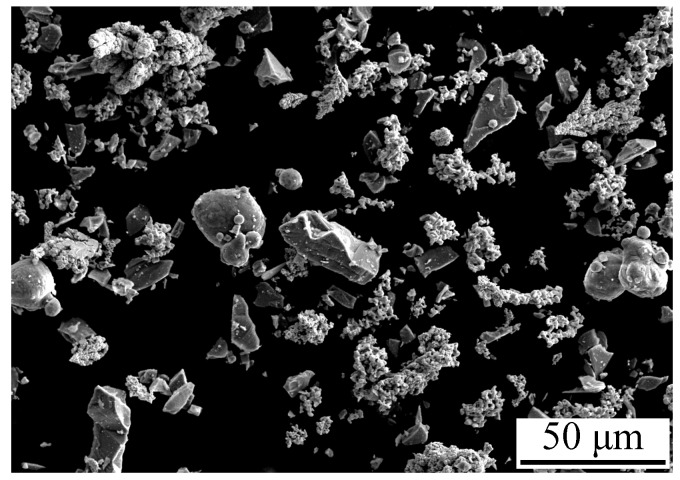
Surface morphology of the mixture of the initial powders for mechanical alloying.

In the present study, the following elemental powders (particle size <75 μm, purity ≥99.5 wt %) were used for mechanical alloying: Al (atomization), Co (reduction), Cr (crushing), Fe (reduction), Ni (electrolysis), and Si (reduction). An equimolar mixture of the initial powders was obtained by mechanical blending prior to mechanical alloying. The surface morphology of the mechanically blended powder mixture is shown in Figure 1.

Mechanical alloying of the AlCoCrFeNiSi HEA powder was carried out at room temperature in a high-energy planetary ball mill (QM-3SP2, NanDa Instrument Plant, Nanjing, China) with 304 stainless steel pots and balls. Mechanical alloying was done for 5, 10, 20, and 30 h with the following operating parameters: ball-to-powder weight ratio (10:1), powder loading (90 g), and rotational speed (300 r⋅min^−1^). To investigate the evolution of powder characteristics such as microstructure, surface morphology, and particle size distribution during mechanical alloying, powder specimens were obtained at different milling times of 5, 10, 20, and 30 h.

### 2.2. Deposition of the AlCoCrFeNiSi HEA Coating

In this study, the powder milled for 10 h was chosen as a feedstock to deposit the coating by atmospheric plasma spraying. Prior to spraying, the feedstock powder was dried in an oven at 200 °C for 3 h to remove the moisture and improve its flowability. First, 316 stainless steel cylindrical specimens with dimensions of Φ 25 × 7 mm were used as substrates. Before spraying, the substrates were cleaned with an ultrasonic cleaner in acetone for 10 min, and then dried with a dryer. To improve the bonding strength of the coating, sand blasting one side of the substrate was carried out to obtain a certain cleanliness and roughness. During sand blasting, an Al_2_O_3_ particle with a size of less than 1.25 mm was used as an abrasive. The substrates were sandblasted under a compressed air pressure of about 0.5 MPa for 30 s. An APS system (3710, PRAXAIR SURFACE TECHNOLOGIES, Indianapolis, IN, USA) with a high-energy plasma gun (SG-100) was employed to deposit the coating. During spraying, argon was used as a primary gas, and nitrogen was used as a secondary gas, whose pressures were 0.4 and 0.3 MPa, respectively. During spraying, the powder was fed into the plasma jet using argon, at a rotating speed of the powder feeder of 0.8 r·min^−1^. The plasma gun traversed in front of the substrate at a spray angle of 90°, a spray distance of 100 mm, and a traverse speed of 200 mm·s^−1^, and the plasma arc power used in the experiment was 45 kW.

### 2.3. Microstructure Characterization

An X-ray diffractometer (XRD, LabX XRD-6000, SHIMADZU, Kyoto, Japan) with Cu–Kα radiation was used to characterize the phase structure of both the mechanically alloyed powders and the APS coating. A 2*θ* angle range of 20–90° and a scanning speed of 3°⋅min^−1^ were used. Jade was used to analyze the phases present in the powders, and measure the average grain size and lattice strain.

A field emission scanning electron microscopy (FESEM, ZEISS ƩIGMA, ZEISS, Oberkochen, Germany) equipped with an energy dispersive spectroscopy (EDS, x-act, OXFORD INSTRUMENTS, Oxford, UK) was used to observe and analyze the morphology and the cross-sectional microstructure of the mechanically alloyed powders, the APS coating, and the wear surface.

Analysis of the grain size and crystal structure of the AlCoCrFeNiSi coating was carried out using transmission electron microscopy (TEM, Tecnai 12, Philips, Amsterdam, The Netherlands). During preparation of the TEM specimens, discs with a diameter of 3 mm were punched from sheets of the coating, and then electropolished in a twin-jet electro polishing device (DJ2000, Beijing Dedong Technology Ltd., Beijing, China). A mixture of absolute alcohol (92 vol %) and perchloric acid (8 vol %) was used as an electrolyte.

### 2.4. Particle Size Measurement of the AlCoCrFeNiSi HEA Powder

Particle size distribution of the mechanically alloyed powders was measured by a laser diffraction sizer (MASTERSIZER 3000, Malvern Instruments Ltd., Malvern, UK). The technique of laser diffraction is used to measure the particle size distribution. In a laser diffraction measurement, a laser beam passes through a dispersed particulate sample, and the angular variation in intensity of the scattered light is measured. Large particles scatter light at small angles relative to the laser beam, and small particles scatter light at large angles. The angular scattering intensity data is then analyzed to calculate the size of the particles that created the scattering pattern using the Mie theory of light scattering. The particle size is reported as a volume equivalent sphere diameter. During the measurement, purified water was used as a dispersant.

### 2.5. Microhardness Test of the APS AlCoCrFeNiSi HEA Coating

For the APS coating, the cross-section was prepared by conventional metallographic techniques. During the measurement, a Vickers microhardness tester (KB 30 S, KB Prüftechnik, Ismaning, Germany) was used with a load of 200 g and a dwell time of 10 s, and the average microhardness value was obtained from more than 10 indentation measurements.

### 2.6. Wear Behavior of the AlCoCrFeNiSi HEA Coating

A high temperature ball-on-disc friction and wear tester (HT-1000, Zhongke Kaihua Science and Technology Development Co. Ltd., Lanzhou, China) was used to investigate the wear behavior of the coating. A Si_3_N_4_ ball with a dimension of Φ 5 mm was fixed on a loading rod. The coating specimen was fixed on a specimen pan by screws. They can contact with each other with the help of the weight with a load of 5 N. During the test, the friction force was continuously measured by a force sensor, and then divided by the normal load to calculate the coefficient of friction (COF). Prior to the test, the coating was polished to reach a roughness of about *Ra* = 0.7 μm. The coating specimen rotated at a set velocity of 573 r·min^−1^, with a friction radius of 5 mm. The coating specimen was taken out when the wear time reached 30 min. The cross-sectional area of the wear track *A* (mm^2^) can be measured by a three-dimensional (3D) confocal laser scanning microscope (LEXT OLS4000, OLYMPUS, Tokyo, Japan), and the volume wear rate *W* (mm^3^·N^−1^·m^−1^) can be calculated according to *W* = *A*·*P*/(*S*·*L*), where *P* is the perimeter of the wear track in “mm”, *S* is the sliding distance in “m”, and *L* is the load applied in “N”. During the experiment, three specimens were tested.

## 3. Results and Discussion

### 3.1. Microstructural Evolution of the AlCoCrFeNiSi HEA Powder during Mechanical Alloying

#### 3.1.1. Surface Morphology and Particle Size

**Figure 2:**
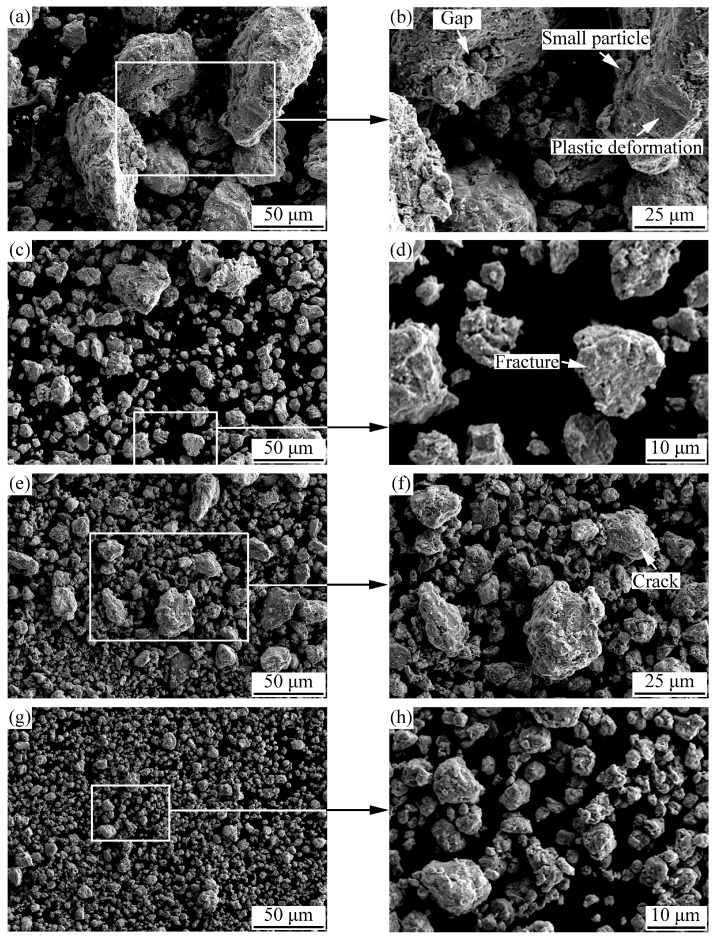
Surface morphology of the AlCoCrFeNiSi powder at different milling times: (**a**,**b**) 5 h; (**c**,**d**) 10 h; (**e**,**f**) 20 h; (**g**,**h**) 30 h.

**Figure 3:**
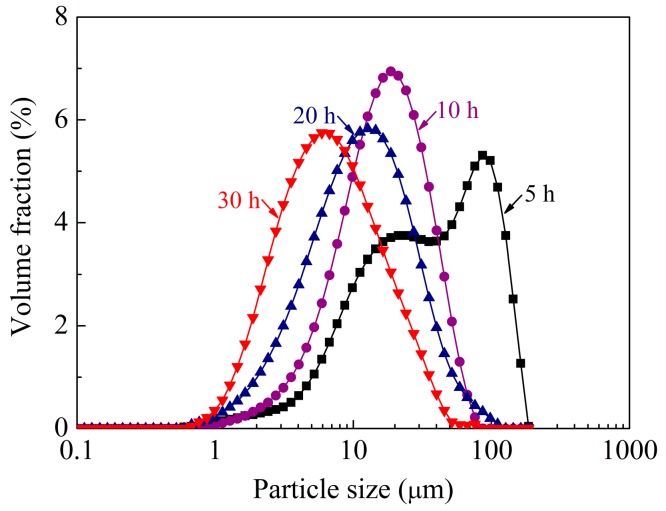
Particle size distribution of the AlCoCrFeNiSi powder at different milling times.

Surface morphology of the AlCoCrFeNiSi powders milled for 5, 10, 20, and 30 h is shown in Figure 2, and their particle size distribution is shown in Figure 3. Significant changes with the milling time can be found upon comparing the morphology and the particle size of the mechanically alloyed powders with that of the mixture of the initial powders in Figure 1.

With the prolonging of the milling time, the shape of the mechanically alloyed powders was closer to spherical. For example, the powder milled for 5 h showed a nearly flat shape (Figure 2a), while the powder that was milled for 10 h showed a near-equiaxed shape (Figure 2c). As the milling time increased to 20 h and then to 30 h, the shape of the powders became closer to spherical (Figure 2e,g). When the powder was milled for 30 h, a nearly spherical shape was formed, which can be observed in Figure 2g. Moreover, at higher magnifications, some details can be found in Figure 2b,d,f,h. From Figure 2b, obvious plastic deformation and some gaps can be seen on the surface of the powder milled for 5 h, and lots of small particles adhered to it, which caused a quite rough surface. With the increase of milling time, small particles adhering to the powder decreased, and the surface became smoother and smoother. In addition, it is noted that fractures and cracks can be observed on the powder surface at milling times of 10 and 20 h, respectively.

From Figure 1, Figure 2 and Figure 3, it can be seen that with the prolonging of the milling time, the particle size of the powders first increased, and then decreased. As mentioned above, the particle size of the mechanically blended powder mixture ranged from several micrometers to about 40 µm (Figure 1), while that of the powder milled for 5 h displayed a bimodal distribution with two peaks (Figure 3), and most of the particles were ones with a size larger than 100 μm (Figure 2a). As the milling time increased to 10 h, the particles larger than 100 μm disappeared (Figure 2c). A narrower peak range—from several micrometers to about 50 μm—was obtained, and the peak moved leftward (Figure 3), which indicates a decrease of the particle size. As the milling time increased to 20 h, lots of particles with a size smaller than 5 μm appeared, which led to a further motion of the peak (Figure 3). When the powder was milled for 30 h, the particle size decreased significantly, and most of the particles were less than 5 μm (Figure 2g and Figure 3).

During mechanical alloying, the flowability of the powders also changed significantly. Due to its near-equiaxed shape and appropriate particle size distribution, the powder milled for 10 h possessed a good flowability, and can be used as a feedstock to deposit a coating by APS. Meanwhile, the flowability of the powder milled for 5 h was limited by its near-flat shape. Although the powders milled for 20 and 30 h had a shape that was nearly spherical, the appearance of the fine particles can also deteriorate their flowability. Therefore, the shape and the particle size distribution of the powders can significantly affect the flowability.

At the initial stage of the MA process (5 h), under the intense impact and rolling of the ball-milling media, plastic deformation of the initial metallic powders with good plasticity occurred, and particles with a flat shape formed. Then, cold welding of the flat particles took place, and the powder with a flat shape in Figure 2a was formed. Therefore, at the initial stage, plastic deformation and cold welding were the dominant processes, which led to an increase of the particle size. The gaps and small particles adhering to the surface (Figure 2b) should be caused by the particles that were not cold welded well, due to the short milling time. With the increase of milling time to 10, 20, and then to 30 h, work hardening occurred because of the repeated deformation of the powder. With the decrease of plasticity, plastic deformation and cold welding processes were weakened, and the fracture process was enhanced, which led to the fractures (Figure 2d) and the cracks (Figure 2f). In addition, the work hardening can also lead to a decrease of the particle size. Besides the variation of the powder morphology and particle size, the particle refinement during mechanical alloying is usually accompanied by atomic diffusion between different elements and formation of solid solution phases [19].

#### 3.1.2. Cross-Sectional Microstructure

**Figure 4:**
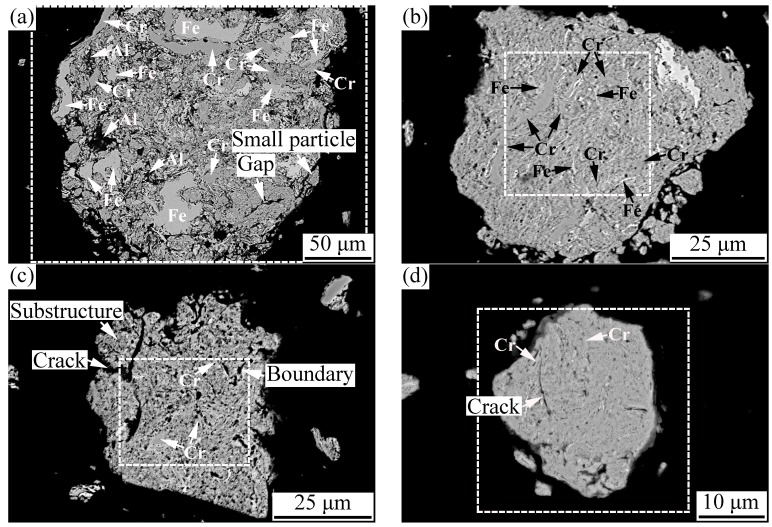
Cross-sectional microstructure of the AlCoCrFeNiSi powder at different milling times: (**a**) 5 h; (**b**) 10 h; (**c**) 20 h; and (**d**) 30 h.

**Figure 5:**
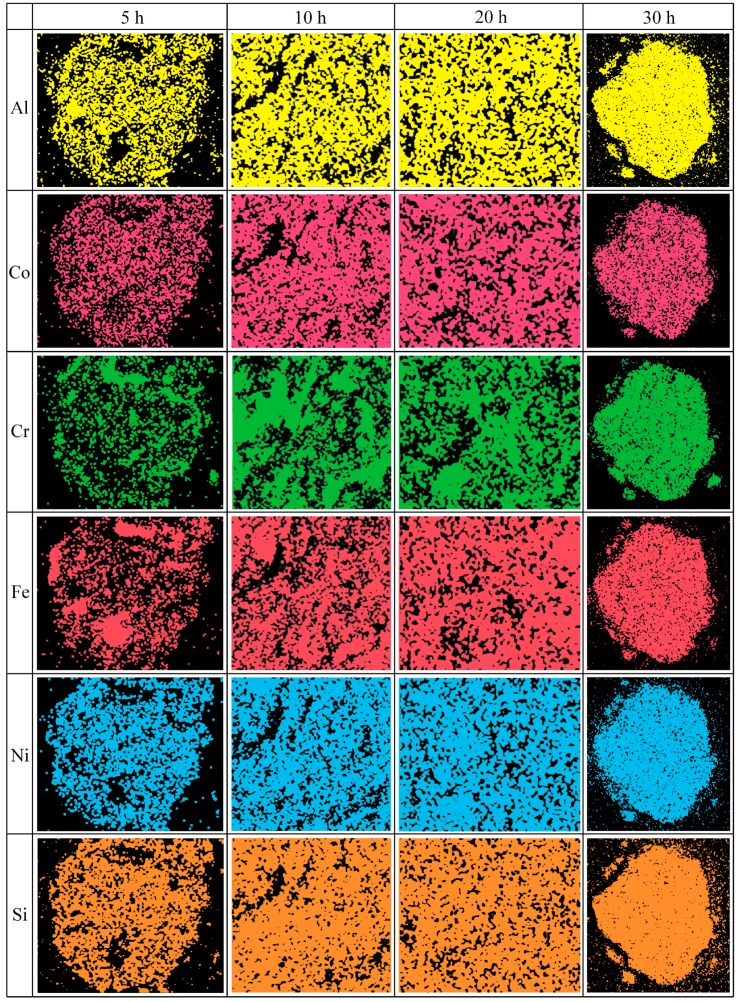
Energy dispersive spectroscopy analysis results of micro-areas on a cross-section of the AlCoCrFeNiSi powder in Figure 4.

The cross-sectional microstructure of the AlCoCrFeNiSi HEA powder at different milling times is shown in Figure 4, and the EDS analysis results of the powders are shown in Figure 5. From Figure 4a, it can be observed that at the initial stage of mechanical alloying (5 h), a loose structure with lots of gaps and small particles adhering to the particle surface was formed, which is consistent with the results in Figure 2b. EDS results (Figure 5) show that some Al particles and lots of Cr and Fe particles were present in the powder, which are marked in Figure 4a. Fe and Cr particles with a flat shape and a thickness of about 10–30 μm were present in the powder, which further indicates that plastic deformation of the particles occurred at the initial stage. In addition, the distribution of Co, Ni, and Si elements was uniform at 5 h (Figure 5).

At the milling time of 10 h, a lamellar structure was formed, and the powder became dense. The Al particles disappeared, and the Al element was distributed uniformly in the powder (Figure 5). Although Fe and Cr particles could still be observed (Figure 4b), their thickness decreased significantly, to a dimension of less than 5 μm. When the powder was milled for 20 h, a finer structure was formed (Figure 4c). Although some Cr particles with a thickness of about 2 μm could still be found, other elemental particles nearly disappeared (Figure 4c and Figure 5). It is noted that lots of substructures were formed in the powder, and some boundaries of substructure and cracks could be seen on the cross-section in Figure 4c. At the milling time of 30 h, the lamellar structure in the powder became much finer and less clear (Figure 4d). The thickness of the Cr elemental particles decreased to a dimension of less than 1 μm, and the distribution of all of the elements became more uniform (Figure 5). In addition, on the cross-section of the powder milled for 30 h, cracks could also be observed (Figure 4d).

Furthermore, it is noted that at the milling time of 5 h, the Si element had been distributed uniformly in the powder, and with a further increase of the milling time to 10 h, 20 h, and then to 30 h, its distribution changed slightly.

**Figure 6:**
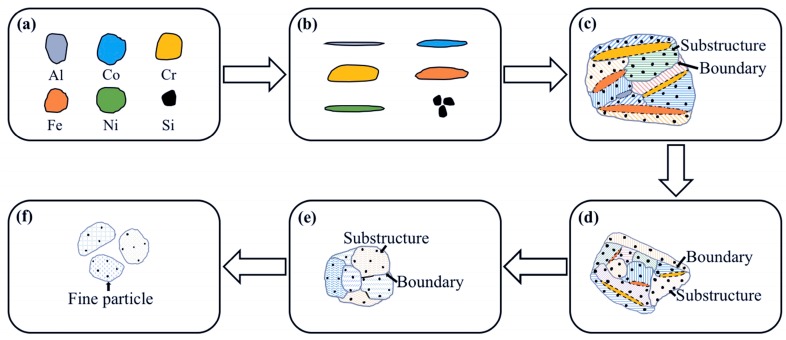
Sketch of the microstructural evolution of AlCoCrFeNiSi powder during mechanical alloying: (**a**) initial particles; (**b**) deformation of the initial particles; (**c**–**e**) structure refinement of the powder; (**f**) fracture of the powder.

During mechanical alloying, the variation regularity of the powder microstructure in this paper is similar with that of the FeAl–TiC ductility–brittleness system in the author’s previous study [27]. As shown in Figure 6, in the AlCoCrFeNiSi system, metallic elements such as Al, Co, Cr, Fe, and Ni exhibit high plasticity and go through plastic deformation, cold welding, and fracture processes, and the elemental size during mechanical alloying was influenced by their hardness and ductility. Due to its cubic diamond structure and strong covalent bonds between atoms, the Si element was difficult to be deformed and cold welded with other elements. It was easily pulverized and embedded into the metallic matrix as elemental particles, and was distributed more and more homogeneously (Figure 6). At the initial stage of the MA process, under the intense impact and rolling of the ball-milling media, plastic deformation of the initial plastic metallic particles changed from near-equiaxed (Figure 6a) to a flat shape (Figure 6b) [28]; then, cold welding of the flat particles took place, which resulted in an intermixing of the elements (Figure 6c,d). However, due to the short milling time, the particles were not well cold welded, which led to a loose structure with lots of gaps (Figure 2 b and Figure 4a). With further prolonging of the milling time to 10, 20, and 30 h, deformation, cold welding, and fracture of the powder proceeded, and the powder became more dense (Figure 4b–d). Meanwhile, with the gradual dissolution of elements during mechanical alloying, the elemental particles decreased, and finally disappeared. The lamellar structure became finer and finer (Figure 4b–d), and at the milling time of 30 h, the lamellar structure was too fine to be identified clearly (Figure 4d). However, because of the repeated deformation of the powder, work hardening occurred. The decrease of the powder plasticity enhanced the fracture process, which led to the fracture of the powder along the boundaries of the substructure (Figure 4c), and the decrease of the particle size (Figure 6f). Finally, as the powder was milled for 30 h, the substructure became finer particles.

#### 3.1.3. X-ray Diffraction

**Figure 7:**
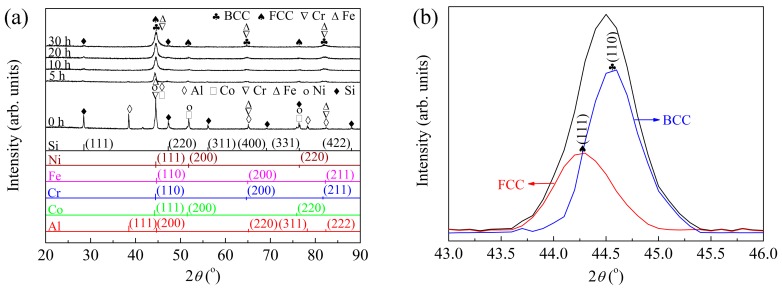
X-ray diffraction results of the AlCoCrFeNiSi powder at different milling times (**a**) and deconvolution of the major peak of the powder milled for 10 h (**b**).

During mechanical alloying, powder specimens milled for 0, 5, 10, 20, and 30 h were obtained and detected by XRD in order to investigate the phase structural evolution, and the results are shown in Figure 7. From Figure 7, it can be found that the mixture of the initial powders was composed of elemental phases of Al, Co, Cr, Fe, Ni, and Si. Compared with the XRD pattern of the powder mixture, that of the powder milled for 5 h changed significantly. A decrease of the diffraction peak intensity of Al, Co, Cr, Fe, Ni, and Si elements can be observed obviously, and the diffraction peaks of Si and Al at about 38° can still be detected, which is consistent with the results shown in Figure 4a and Figure 5.

When milled for 10 h, the diffraction peak of the Al element at about 38° disappeared, while those of the Si element could still be observed. A significant broadening of the major peak at about 45° took place, which indicates that solid solution phases were formed [22,29]. When the major peak was enlarged (Figure 7b), it was found to be asymmetric, and a slight shoulder at about 44.3° could be observed. After deconvolution, a minor FCC phase with maximum intensity at 44.3°, and a major BCC phase with a maximum intensity at 44.6° were found. Therefore, besides the Cr and Fe detected in Figure 4b and Figure 5, FCC and BCC solid solution phases were formed as new phases. It was reported that materials with higher melting points generally possess higher bonding energies between atoms, resulting in lower diffusion coefficients [20]. As listed in Table 1, the Cr element, which has a high melting point, higher hardness, and a lower self-diffusion coefficient, is limited to serving as a solvent during mechanical alloying. Therefore, in this study, the Fe element should serve as the solvent during the formation of the BCC solid solution, which is corresponding with the report [20]. After measurements from the (110) peak corresponding to the BCC phase in Figure 7b, an average grain size of 11.7 nm with a lattice strain of about 0.73% was obtained.

With the increase of milling time to 20 h and then to 30 h, no obvious changes of the diffraction peaks can be seen in Figure 7a. It is noted that the diffraction peaks of the Si element were still visible when the powder was milled for 30 h. As it is similar with Cr, the Si element, with its strong Si–Si covalent bond, cubic diamond structure, higher hardness, and lower self-diffusion coefficient, is also limited to serve as a solvent and a solute during mechanical alloying.

The formation of the solid solution phase is usually attributed to the following factors: the chemical mixing enthalpies of atomic pairs near zero, the small electronegativity difference between the elements, a relative atomic size difference less than 15% [20], and a high mixing entropy. As listed in Table 2, most atomic pairs possess quite low enthalpies. For example, the enthalpies of atomic pairs between Si/Al and other elements are much lower than zero, which can limit the formation of the solid solution. However, electronegativity between the elements possesses a small difference [34,35], which is beneficial to the formation of a metallic bond instead of a non-metallic bond [20]. Meanwhile, the lower relative atomic size difference is also beneficial to the formation of the solid solution phase. For the AlCoCrFeNiSi system, the mixing entropy calculated according to the Boltzmann’s hypothesis [1,36] was as high as about 15 J·K^−1^·mol^−1^, which can also accelerate the random diffusion of different elements.

### 3.2. Characterization of the APS AlCoCrFeNiSi HEA Coating

#### 3.2.1. Surface Morphology

**Figure 8:**
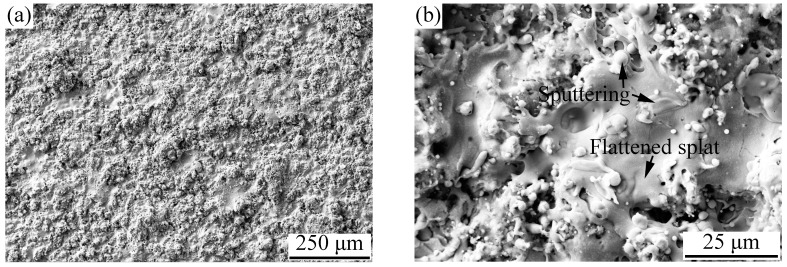
Surface morphology of the AlCoCrFeNiSi coating at magnifications of 100× (**a**) and 1000× (**b**).

In this study, because of its appropriate particle size distribution and good flowability, the powder milled for 10 h was chosen as a feedstock to deposit a coating. Figure 8 shows the surface morphology of the APS AlCoCrFeNiSi HEA coating. From Figure 8a, it can be seen that the coating showed a rough morphology, which is usually observed on the surfaces of atmospheric plasma sprayed coatings [9]. It can be observed that at a higher magnification (Figure 8b), most of the splats were fully molten and well flattened, and the outlines of the flattened splats could be observed obviously. Some spherical and droplet-shaped particles with dimensions of less than 10 μm could also be found on the coating surface, which originated from the sputtering of the flying particles when impacting the already formed coating surface.

#### 3.2.2. Cross-Sectional Microstructure

**Figure 9:**
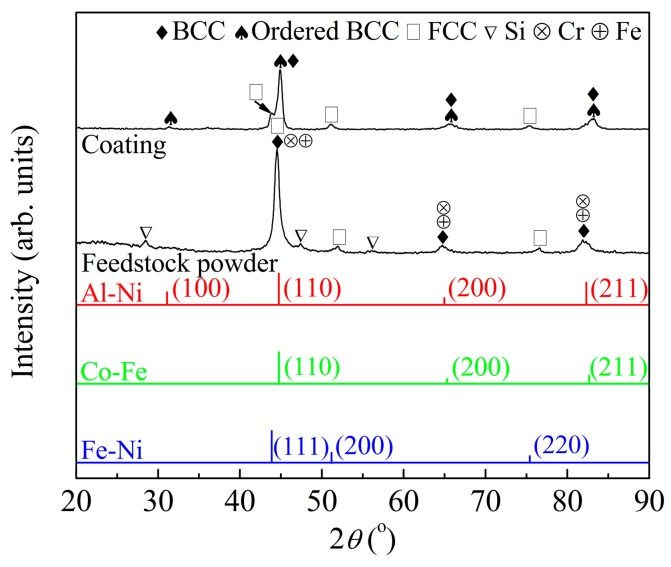
X-ray diffraction results of the AlCoCrFeNiSi coating and the feedstock powder.

Figure 9 shows the XRD results of both the APS coating and the feedstock powder. Obvious difference can be found upon comparing the XRD pattern of the coating with that of the feedstock powder. As mentioned above (Figure 7), the powder milled for 10 h was composed of BCC, FCC, and a small amount of Fe, Cr, and Si elemental phases. After plasma spraying, besides the matrix BCC phase, an ordered BCC phase was detected. In addition, the peak intensity of the FCC phase increased, while the diffraction peaks of the Si elemental phase disappeared. These results indicate that phase transformation occurred during plasma spraying.

**Figure 10:**
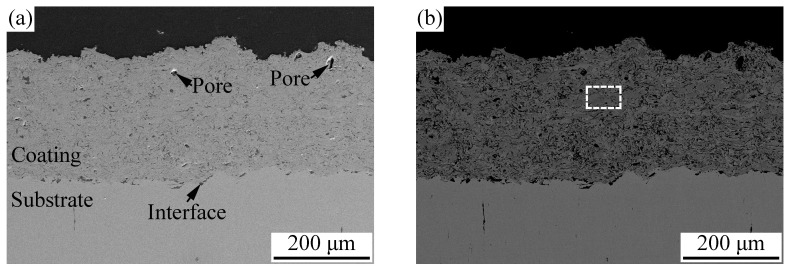

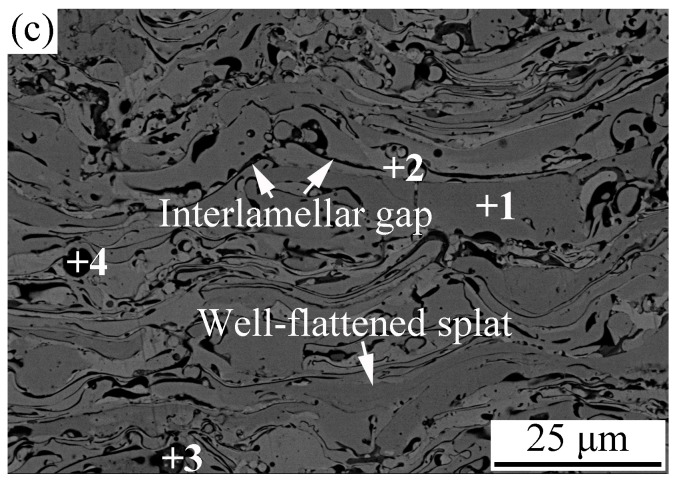
Cross-sectional microstructure of the AlCoCrFeNiSi coating at magnifications of 150× in a secondary electron mode (**a**) and a back-scattered electron mode (**b**) and 1000× (**c**).

**Figure 11:**
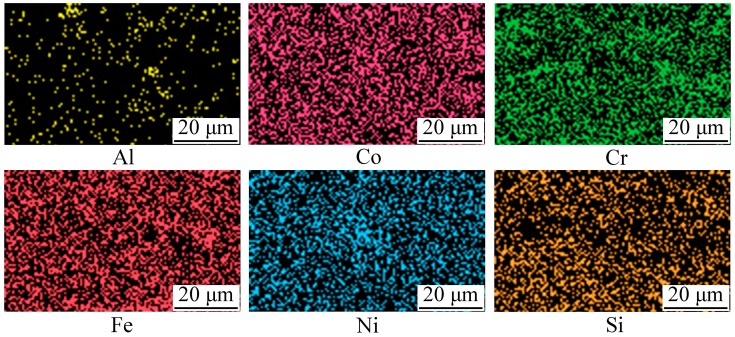
Energy dispersive spectroscopy (EDS) elemental mapping of the AlCoCrFeNiSi coating in a randomly selected area in Figure 10b.

Figure 10 shows the cross-sectional microstructure of the APS AlCoCrFeNiSi coating. From Figure 10a,b, it can be seen that a coating with a thickness of about 220 μm was obtained. Besides a few obvious near-spherical pores with dimensions of less than 10 μm, the microstructure of the coating was dense and uniform. A good bonding was formed between the coating and the substrate. After sand blasting, a rough surface of the substrate was formed, and the undulating outline can be observed in Figure 10a,b. Besides limited pores with dimensions of several micrometers, the bonding between the coating and the substrate was continuous, and mechanical occluding was formed between the splats and the rough substrate surface. It can be observed from Figure 10c that most of the splats were fully molten and well flattened, which can also be found on the coating surface (Figure 8b). Some unmelted and semi-melted particles were also present in the coating. Generally, during atmospheric plasma spraying, heating of the plasma jet on the flying particles is stochastic. When the feedstock powder with a wide particle size range is injected into the plasma jet, different kinetic and thermal energy is obtained by the particles, which can cause different states. Fully molten particles with enough velocity will form the well-flattened splats, while unmelted or semi-molten particles will rebound or form the unmelted and semi-molten particles in the coating. In Figure 10c, all of the flattened splats and unmelted particles were mechanically bonded with each other, and a typical lamellar structure was formed. Comparing with the cross-section of the powder milled for 10 h (Figure 4b), the lamellar structure inside a single particle disappeared, and a splat with a uniform structure was formed, which indicates that elemental rearrangement took place after plasma spraying. During high-temperature spraying, as the mechanically alloyed powder is injected into the plasma jet, molten droplets with a uniform chemical composition are formed from the particles. When the droplets impact the substrate or the previously formed splats, cooling and solidification occur, and finally, splats with a uniform structure are formed. To further investigate the elements distribution of the AlCoCrFeNiSi coating, an EDS elemental mapping was carried out in a randomly selected area in Figure 10b. The results in Figure 11 show that after spraying, the elements of Al, Co, Cr, Fe, Ni, and Si were distributed uniformly in the coating.

At a higher magnification (Figure 10c), areas with different contrasts can be observed. EDS analysis was carried out to determine the composition of some typical areas, and the results are listed in Table 3. Results show that the matrix such as point 1 was rich Al, Fe, Co, and Ni elements, which should be corresponding to the Al–Ni ordered BCC and Co–Fe BCC phases, according to the XRD result in Figure 9. It was reported that the segregation of the Al and Ni elements was due to the negative mixing enthalpy between Al and Ni in the alloy system [38], which can facilitate the formation of precipitation of Al–Ni ordered BCC phase. Point 2, which had a light gray contrast, was rich with Fe and Ni elements, except for the Al element, and it should have corresponded to a Fe–Ni solid solution with a FCC structure (Figure 9). Some points with dark gray contrasts (such as 3 and 4) were rich Cr, Al, and oxygen elements, which can be caused by the oxidation of Cr and Al elements in the high-entropy alloy powders. During plasma spraying, oxygen in the surrounding air is usually interfused into the plasma jet and reacts with the high-temperature droplets, after which the oxides in the coating are finally formed.

**Figure 12:**
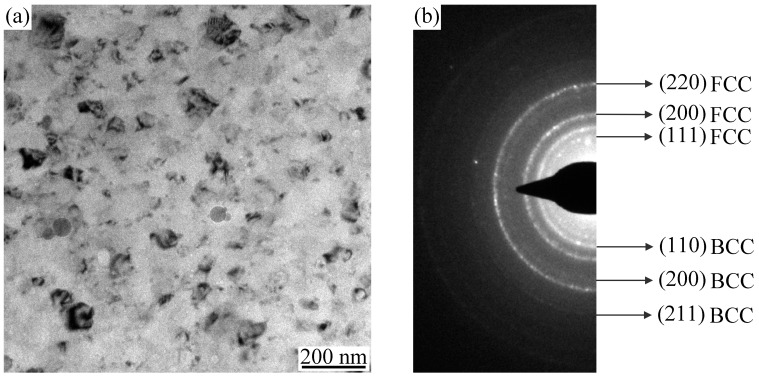
Bright field transmission electron microscopy image of the AlCoCrFeNiSi coating (**a**) and the selected area of the electron diffraction ring pattern corresponds to the body-centered cubic (BCC) and face-centered cubic (FCC) phases (**b**).

To further investigate the microstructure and phase structure of the AlCoCrFeNiSi coating, TEM analysis was performed, and the bright field image and the selected area electron diffraction (SAED) ring pattern are shown in Figure 12. From the TEM image in Figure 12a, nanometer crystalline grains with a grain size of about 30 nm can be observed in the coating. The SAED result shows that the coating has a polycrystalline structure, which is composed of BCC and FCC structures (Figure 12b).

#### 3.2.3. Microhardness and Wear Behavior

**Figure 13:**
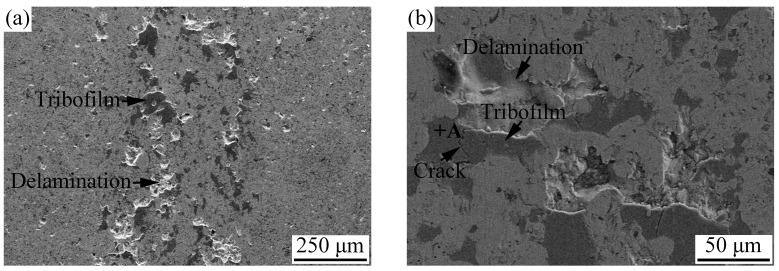

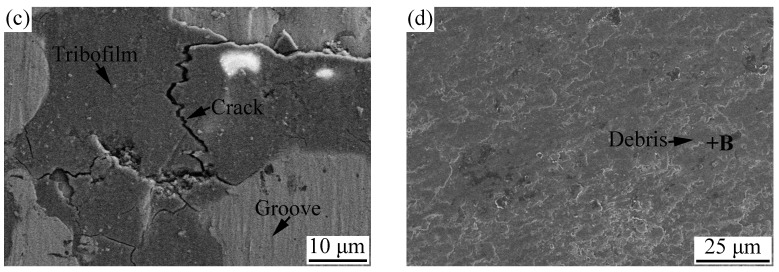
Wear surfaces of the AlCoCrFeNiSi HEA coating (**a**–**c**) and wear scar on the surface of the counterpart (**d**).

The microhardness of the APS AlCoCrFeNiSi HEA coating was measured, and an average value of 612 ± 41 HV was obtained. Usually, coatings with high hardness exhibit good wear resistance. The wear surfaces of the AlCoCrFeNiSi HEA coating and the wear scar on the surface of the counterpart are shown in Figure 13. From Figure 13a,b, some delamination of splats at local areas on the wear surface can be found, and flaky debris can be observed on the counterpart surface in Figure 13d. These results illustrate that adhesive wear was the main wear mechanism. For thermally sprayed coatings, a limited interlamellar bonding ratio (less than one third) was found between splats, which can deteriorate their mechanical properties, such as bonding strength and wear resistance [27]. In this study, during the wear test, as the delamination of the non-bonded area of the splats took place and flaky debris on the counterpart surface was formed, material transferred from the coating to the Si_3_N_4_ ball.

In addition, lots of discontinuous areas with a dark gray contrast could be found on the wear surface (e.g., point A in Figure 13b), and the EDS analysis result listed in Table 4 shows that a high oxygen content was detected in this area. It was reported that these areas, which were defined as tribofilms, can be caused by the flash temperature at the contact points between the surfaces of the coating and the counterpart [39]. In addition, cracks across the tribofilms can be found in Figure 13b,c, which resulted from the fracture of the brittle tribofilms with the load applied. Besides, an oxygen element with a high content was also detected on the wear scar of the counterpart, e.g., point B in Figure 13d (Table 4). All of the above results indicate that tribo-oxidation wear took place during the test.

From Figure 13c, fine grooves along the sliding direction can be observed in the area without tribofilms, while no grooves were found on the hard tribofilms. During the wear test, when the Si_3_N_4_ ball slid on the coating surface, the hard debris on the ball surface acted as ploughs, and the grooves were formed. Therefore, slight abrasion wear also took place during the test.

**Figure 14:**
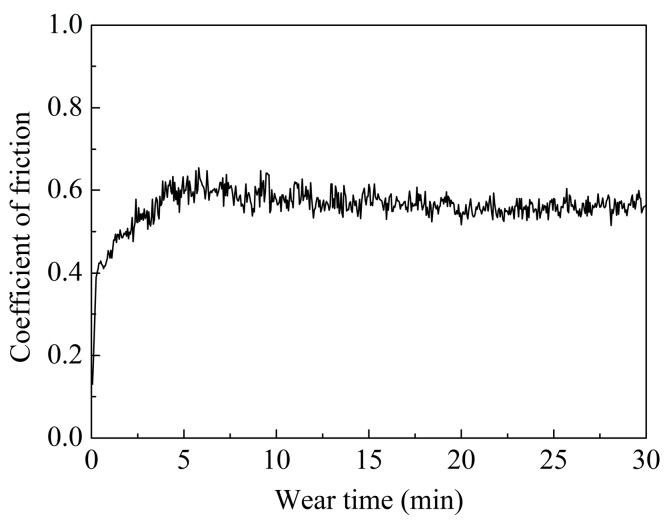
Coefficient of friction of the AlCoCrFeNiSi HEA coating with the change of the wear time.

The coefficient of friction of the AlCoCrFeNiSi HEA coating with the change of the wear time is shown in Figure 14, from which an obvious running-in stage in the initial 6 min, and a following steady wear stage, can be observed. At the beginning of the running-in stage, the COF curve showed a lower value, which can be attributed to the polished coating surface with a lower roughness. With an increase of the wear time, the appearance of the discontinuous tribofilms, delamination, cracks, and grooves on the wear surface (Figure 13), which can result in a higher roughness, caused an increase of the COF. At the steady wear stage, an average value of 0.57 with a standard deviation of 0.02 was obtained. Compared with the AlCoCrFeNiTi coating with an average COF of 0.82 ± 0.05 in our previous study [18], the AlCoCrFeNiSi coating showed a lower value, which indicates that the coating in this study exhibited better wear resistance.

The AlCoCrFeNiSi HEA coating showed a volume wear rate of (0.38 ± 0.08) × 10^−4^ mm^3^·N^−1^·m^−1^, which is about half that of the AlCoCrFeNiTi coating in our previous study (0.77 ± 0.01) × 10^−4^ mm^3^·N^−1^·m^−1^ [18] at the same test conditions. This result indicates that the wear resistance of the AlCoCrFeNiSi coating was better than that of the AlCoCrFeNiTi coating. Besides, comparing with the conventional flame-sprayed NiCrBSi coating with a volume wear rate of about 1.7 × 10^−4^ mm^3^·N^−1^·m^−1^ [40], the coating in this paper also showed much better wear resistance. All of these results make the APS AlCoCrFeNiSi coating a promising one that can be used in abrasion environments in the industries of petroleum, power, mining, and so on. Besides the high density of the coating and the high hardness of the AlCoCrFeNiSi material, formation of the hard tribofilms (Figure 13) can be another reason for the good wear resistance.

## 4. Conclusions

At the milling time of 10 h, a simple BCC solid solution with a Fe element serving as a solvent, and FCC solid solution phases were formed.

An AlCoCrFeNiSi HEA coating can be deposited by APS using the powder milled for 10 h. Compared with the mechanically alloyed powder, the lamellar structure inside a single particle disappeared, which indicates that elemental rearrangement took place during plasma spraying. After plasma spraying, an ordered BCC phase was detected, and the diffraction peaks of the Si element disappeared, which indicates that phase transformation occurred during plasma spraying. TEM analysis result shows that nanometer crystalline grains with a grain size of about 30 nm existed in the as-sprayed coating.

An average microhardness of 612 ± 41 HV was obtained for the APS AlCoCrFeNiSi coating. Adhesive wear, tribo-oxidation wear, and slight abrasion wear took place during the wear test. The coating showed good wear resistance, with a volume wear rate of (0.38 ± 0.08) × 10^−4^ mm^3^·N^−1^·m^−1^, which indicates that it can be used in abrasion environments in fields of petroleum, power, mining, and so on.

## Figures and Tables

**Table 1:** Properties of the related elements [20,30,31,32,33].

Element	Al	Co	Cr	Fe	Ni	Si
Atomic radius (Å)	1.43	1.25	1.25	1.24	1.24	1.18
Melting point (°C)	660.3	1454.9	1856.9	1534.9	1452.9	1409.9
Crystal structure	FCC	HCP	BCC	BCC	FCC	Cubic diamond
Electronegativity *χ*_a_	1.61	1.88	1.66	1.83	1.91	1.90
Vickers hardness (HV)	130	310	1060	160	172–184	1300 ± 100
Self-diffusion coefficient at 27 °C (mm^2^·s^−1^)	10^−23^	10^−48^	10^−53^	10^−40^	10^−47^	10^−81^

**Table 2:** Chemical enthalpy of mixing (Δ*H*^mix^) of binary equiatomic alloys between the related elements calculated by Miedema’s model (kJ·mol^−1^) [30,37].

Element	Al	Co	Cr	Fe	Ni	Si
Al	-	−19	−10	−11	−22	−19
Co	-	-	−7	−1	0	−38
Cr	-	-	-	−1	−7	−37
Fe	-	-	-	-	−2	−35
Ni	-	-	-	-	-	−40

**Table 3:** Energy dispersive spectroscopy analysis results of typical points on cross-section of the AlCoCrFeNiSi coating in Figure 10c (at %).

Point	Al	Co	Cr	Fe	Ni	Si	O
1	17.4	21.7	10.3	11.6	20.7	18.3	0.0
2	0.0	13.7	15.1	33.2	32.8	5.2	0.0
3	5.4	2.9	35.5	3.6	2.2	2.6	47.7
4	42.0	0.4	0.4	0.0	0.4	0.0	56.8

**Table 4:** Energy dispersive spectroscopy (EDS) analysis results of typical points on the wear surfaces of the AlCoCrFeNiSi high-entropy alloy (HEA) coating, and the wear scar on the surface of the counterpart in Figure 13 (at %).

Point	Al	Co	Cr	Fe	Ni	Si	O	N
A	4.16	4.84	5.40	4.86	5.11	5.91	69.72	0.00
B	1.80	0.00	0.00	0.00	0.00	11.05	56.22	30.92
